# Psychometric findings for LIMB-Q kids based on an international study of 800 children and adolescents with lower limb differences

**DOI:** 10.1186/s41687-025-00916-y

**Published:** 2025-07-05

**Authors:** Harpreet Chhina, Charlene Rae, David Podeszwa, James A. Fernandes, Louise Johnson, Bjoern Vogt, Jan Duedal Rolfing, Alicia Kerrigan, Ashish Ranade, Juergen Messner, Mohan Belthur, Melissa Esparza, Jonathan Wright, David Bade, Sarah McMahon, Christopher Iobst, Sanjeev Sabharwal, Marcel Abouassaly, Anthony Cooper, Anne F. Klassen

**Affiliations:** 1 Department of Orthopaedics, University of British Columbia, Vancouver, BC Canada; 2 Department of Pediatrics, McMaster University, Hamilton, Ontario Canada; 3 Department of Orthopaedics, Scottish Rite for Children, Dallas, Texas USA; 4 Department of Orthopaedics, Sheffield Children’s Hospital, Sheffield, UK; 5 Department of Clinical & Health Psychology, Leeds Teaching Hospitals NHS Trust, Leeds, UK; 6 University of Münster, Münster University Hospital, Münster, Germany; 7 Children’s Orthopaedics and Reconstruction, Aarhus University Hospital, Aarhus, Denmark; 8 Children’s Hospital of Eastern Ontario, University of Ottawa, Ottawa, Ontario Canada; 9 Blooming Buds Centre for Pediatric Orthopaedics, Deenanath Mangeshkar Hospital, Pune, India; 10 Royal Hospital for Children and Young People Edinburgh, Royal Infirmary Edinburgh, Edinburgh, UK; 11 Department of Orthopaedics, Phoenix Children’s Hospital, Phoenix, Arizona USA; 12 Royal National Orthopaedic Hospital, Stanmore, UK; 13 Department of Orthopaedics, Queensland Children’s Hospital, Queensland, Australia; 14 Great Ormond Street Hospital for Children, London, UK; 15 The Ohio State University, College of Medicine, Nationwide Children’s Hospital, Columbus, Ohio USA; 16 UCSF Benioff Children’s Hospital, Oakland, California USA; 17 Alberta Children’s Hospital, Calgary, Alberta, Canada; 18 Department of Orthopaedics, BC Children’s Hospital, University of British Columbia, Vancouver, BC Canada; 19 1D 18, Orthopaedics Research Office, BC Children’s Hospital, 4480 Oak Street, Vancouver, BC V6H 3V4 Canada

**Keywords:** Limb differences, Limb deformities, Quality of life, Health-related Quality of Life, Patient-reported outcomes, Limb reconstruction, Psychometrics, International study

## Abstract

**Background:**

LIMB-Q Kids is a new patient-reported outcome measure (PROM) for children and adolescents with Lower limb differences (LLDs). This international field test study aimed to evaluate the psychometric properties of LIMB-Q Kids.

**Methodology:**

Patients from pediatric orthopaedic clinics with a diagnosis of LLDs were recruited. Participants completed LIMB-Q Kids and 2 generic quality of life questionnaires, i.e., PROMIS Pediatric Short Form v2.0 – Mobility 8a, and PedsQL. Demographic and clinical data were collected including the LLRS AIM Index, a measure of clinical severity for LLDs. Rasch measurement theory (RMT) analysis was used to examine the psychometric properties of LIMB-Q Kids. Test-retest (TRT) reliability was examined and tests of construct validity were performed.

**Results:**

Participants (N = 800) were recruited from 16 sites in 7 countries. Participants were aged 8 years and older (mean = 13, standard deviation = 3.2, range 8–25 years) and had a broad range of LLDs (e.g., Leg Length Discrepancy, Fibular Hemimelia, Skeletal Dysplasia, Blount’s disease, Posteromedial Tibial Bowing, Osteogenesis Imperfecta, Congenital Pseudarthrosis of Tibia, Tibial Hemimelia and Amputations). RMT analysis provided evidence of the reliability and validity of 9 independently functioning scales that measure leg appearance, physical function, symptoms (hip, leg, knee, ankle, and foot), leg-related distress, and social, and psychological function. In addition, TRT reliability based on a sample of 46 participants was high for all 9 scales (Intraclass correlation coefficient ranges from 0.76–0.95). LIMB-Q Kids Physical Function scale correlated strongly with the PROMIS Pediatric Short Form v2.0 – Mobility 8a (Pearson correlation 0.82) and the PedsQL Physical Function total score (Pearson correlation 0.77). As hypothesized, participants with more severe LLDs based on the LLRS AIM index scores reported lower scores on all LIMB-Q Kids scales, indicating more impact on the patients.

**Conclusions:**

This study provided evidence for the validity and reliability of LIMB-Q Kids. This new PROM can be used to inform research, quality improvement efforts, and clinical care. By measuring outcomes that matter most to children and adolescents with LLDs, LIMB-Q Kids can provide information to support evidence-based decisions.

**Level of evidence:**

Level III

**Supplementary Information:**

The online version contains supplementary material available at 10.1186/s41687-025-00916-y.

## Background

Lower limb differences (LLDs) can result from congenital conditions or are acquired from trauma, infection, tumors, or other medical conditions. LLDs mainly include leg length discrepancy, lower limb deficiency, and associated angular and rotational deformities [1]. Children with LLDs can experience physical limitations, gait problems, and pain. Abnormal leg appearance and function can discourage participation in recreational and leisure activities and lead to behavioral, emotional, psychological, and social adjustment problems [2–6]. Treatment options vary widely from non-surgical approaches to complex surgical procedures that can impact health-related quality of life (HRQL) of these patients [6–8]. Non-surgical treatments include the use of adaptive devices, prostheses, orthotics, and shoe lifts. Surgical treatments include reconstructive or ablative surgery along with the use of prostheses and orthotics. The most common surgical procedures for lengthening and deformity correction involve the use of an internal nail or an external fixator. Treatment involving an external fixator has a high complication rate [9]. While the treatment using an internal nail has some advantages over an external fixator, this approach cannot be applied to all patients due to clinical indications [10]. Amputation, though irreversible, can improve function and HRQL when limb salvage is not possible [11]. However, amputation can cause stump overgrowth, pain, and psychological adjustment issues. Currently, there is a lack of evidence to support one treatment approach over another [12–14]. To facilitate future comparative studies of amputation versus limb reconstruction, incorporating a patient-reported outcomes measure (PROM) would provide the patient perspective in outcome assessment.

LIMB-Q Kids is a new PROM for children and adolescents with LLDs. Our systematic review identified the need for a comprehensive PROM for children and adolescents with LLDs [15]. Our goal was to develop a PROM that measures concepts that matter the most to children and adolescents with LLDs and is applicable internationally [16]. A mixed method, multiphase approach, based on international guidelines for PROM development, was followed to develop LIMB-Q Kids [17–20]. A systematic review helped develop a preliminary conceptual framework of concepts that have been studied for this patient population in the past [15]. Three health concepts including physical, psychological, and social health, along with 15 sub-concepts were identified in the systematic review. Concept elicitation interviews were conducted with 79 patients aged 8 to 18 years with a diagnosis of LLD and their parents [21]. Qualitative analysis led to the development of a final conceptual framework of concepts important to this patient population including appearance, physical health, psychological health, school and social health. A pool of items was generated that were used to create a set of independently functioning scales. These scales were further refined in a content validation study including input from 23 experts from Australia, Canada, Ethiopia, India, UK, and the USA. Cognitive debriefing interviews were conducted with 17 patients between the ages of 8–18 years from sites in Australia, Canada and the USA [22]. Five rounds of input was obtained from children, parents, and experts. 37 new items were added based on this study. Thirty-three of the 37 new items added were included to measure symptoms experienced in different parts of the leg. This study focused on three key components of content validity, i.e., comprehension, comprehensiveness, and relevance.

After the content validation study, the field test version of LIMB-Q Kids consisted of 159 items that formed 11 scales, i.e., Leg Appearance, Physical Function, Symptoms (hip, knee, ankle, foot, and leg), Leg-related Distress, and School, Social, and Psychological Function. In preparation for the international field test, LIMB-Q Kids was translated and culturally adapted (TCA) into Danish, German, and Hindi following international guidelines by The Professional Society for Health Economics and Outcomes Research [20, 23–25]. TCA process included two independent forward translations, reconciliation of the two forward translations into one forward translation and back translation by a third translator. This back-translated version was compared with the original English translation by the developers of LIMB-Q Kids (HC) and edits were made as required. An expert panel meeting including healthcare professionals from the sites leading the translations and developers of LIMB-Q Kids (HC) discussed the translated versions to ensure that concepts of interest are adequately reflected in the translated versions. This was followed by cognitive debriefing interviews with 8–10 patients with LLDs, between the ages of 8–18 years, who are native speakers of that language. A final translated version was produced at the end of the cognitive debriefing interviews after incorporating the feedback from the interviewed children. This version was proofread and finalised for use in the field test study.

The aim of this international field test study was to evaluate the psychometric properties (reliability and validity including construct validity) of the LIMB-Q Kids in patients with a diagnosis of LLD recruited from pediatric orthopaedic centers.

## Methods

### Ethics

Institutional ethics approval for this study was obtained at the lead study site (Children’s and Women’s Research Ethics Board, H21-02204), and each participating site’s research ethics board. Data transfer agreements were signed with each participating site. Based on each site’s research ethics board requirements, patients and parents provided written or verbal consent.

### Study participants

Patients aged 8 years and older with a diagnosis of LLDs treated at a pediatric orthopaedic center who were able to read and write in Hindi, Danish, German, or English were recruited from 16 pediatric orthopaedic clinics in Australia (1 site), Canada (3 sites), Denmark (1 site), Germany (1 site), India (1 site), United States (4 sites) and United Kingdom (5 sites). Patients with a medical condition that limited their ability to read or write independently were excluded.

### Data collection

Data collection took place between August 2021 and January 2024. Data were collected during routine clinic visits using 2 main approaches based on each site’s logistics and available resources. One approach was the use of tablets/iPads where data were entered directly by the study participants into a Research Electronic Data Capture (REDCap) database hosted at the first author’s institution in Canada [26, 27]. The second approach was the use of paper booklets by patients, with data entered into the REDCap database by a research assistant from the collaborating site.

Data collection in REDCap included demographic and clinical questions followed by LIMB-Q Kids and 2 generic questionnaires, i.e., the Patient-Reported Outcomes Measurement Information System® (PROMIS) Pediatric Short Form v2.0 – Mobility 8a, and the Pediatric Quality of Life Inventory^TM^ (PedsQL). Participants were asked to provide their age, gender, language spoken, school grade, and whether they go to school in-person or online. Only participants aged 8 to 18 years who went to school with other children were included in the analysis for the School scale. All other LIMB-Q Kids scales included data from the entire sample. At the end of the survey, participants in English-speaking countries were asked to provide their email if they were willing to complete LIMB-Q Kids again in 2 weeks for a test-retest (TRT) study. This 2 week time period was selected based on the COnsensus-based Standards for the selection of health Measurement Instruments (COSMIN) criteria [28].

A clinical form was completed and entered into REDCap by staff at each site. The clinical form asked for patient’s biological sex, hospital name, country of residence, diagnosis, clinical symptoms, and treatment information, including the reason for treatment, type of treatment, stage of treatment, and future treatments planned. The sites also provided the data needed to compute the LLRS AIM Index score, including the location of the deformity, the length of the leg inequality, presence of risk factors, soft tissue injury, angular deformity, infection or bone quality, and motion or stability of the joint [29]. The LLRS AIM Index classifies the patients into minimal, moderate, substantial, and high complexity based on their calculated LLRS AIM Index score (0 = normal, 1–5 = minimal complexity, 6–10 = moderate complexity, 11–15 = substantial complexity,16–28 = high complexity). Clinical teams at the participating sites also provided information on whether the participants had any symptoms in the hip, leg, knee, foot, and ankle. This information was used to examine the floor and ceiling effects for the symptom scales.

The field test version of LIMB-Q Kids consisted of 159 items that formed 11 scales, i.e., Leg Appearance, Physical Function, Symptoms (leg, hip, knee, ankle and foot), Leg-related Distress, and School, Social, and Psychological Function. Each scale included 4 response options. Ten scales used a recall period of 1 week. The exception was the Leg Appearance scale that asked participants to think of how their leg looks at the time of completing the scale.

PROMIS Pediatric Short Form v2.0 – Mobility 8a is a generic instrument that measures physical function and mobility in children aged 8 to 17 years [30]. This PROM has 8 items with 5 response options and uses a 7-day recall period. Participants were also asked to complete the 23-item PedsQL generic measure of HRQL. There are separate modules for children aged 8 to 12 years, adolescents aged 13 to 18 years and youth 18–25 years. These modules measure Physical, Emotional, Social, and School Functioning [31]. The PedsQL has 6 response options and a recall period of one month. Both PROMIS and PedsQL have been used to measure outcomes in pediatric conditions that involve the spine and hip (e.g., fractures and syndactyly, clubfoot, and limb deformities) [6, 13, 32–37].

### Statistical analysis

Rasch measurement theory (RMT) analysis was used to examine the fit of the observed data to the Rasch model for each LIMB-Q Kids scale [38–40]. RMT scales provide interval level measurement, which allows to accurately measure change over time [41]. A scale based on the RMT analysis provides person estimates that are independent of the sampling distribution of the items. RMT allows for differential item functioning (DIF) testing, ensuring that items function equally across groups (e.g., age, gender, or cultural backgrounds). RMT produces person location (ability) and item difficulty on a common logit scale allowing PROM scores to be interpreted linearly, making it easier for clinicians to track meaningful changes.

RMT analysis was conducted in RUMM2030 software (RUMM Laboratory, Perth, WA, Australia) using an unrestricted partial credit model for polytomous data. A range of tests and criteria were conducted to identify the best set of scale items to retain in each scale. The goal was to identify the items that provided a well-targeted scale with high reliability and validity. The psychometric tests that were conducted are shown in Table 1. These tests were considered together to make decisions about item retention [38, 39, 42].

**Table 1:** Psychometric tests performed

Test	Description
Thresholds for item response options	We first examined threshold ordering by visually inspecting the threshold map for each scale. We examined thresholds between response options to determine if a scale’s response categories were ordered, meaning that “1”on a 4-point scale must sit lower in the continuum than a “2”, and so on [41]. This approach is used to create a hierarchy of items to determine how items are ordered from easiest to hardest to endorse. Scores are re-coded when disordered thresholds are seen.
Item fit statistics	Examines the extent to which observed data aligns with expected values based on the Rasch model. Three fit indicators were examined: log residuals (item–person interaction), chi-square (Χ2) values (item–trait interaction), and item characteristic curves. Ideal fit residuals are between – 2.5 and + 2.5 with Χ2 values non-significant after Bonferroni adjustment. These fit statistics are interpreted together in the context of their clinical usefulness. Item characteristic curves can be viewed graphically. The sample was adjusted to 500 for tests of statistical fit.
Reliability	This measurement property examines the accuracy of scores for a scale. Reliability coefficients greater than or equal to 0.70 were considered adequate [43]. We explore three types of reliability: Person Separation Index: This statistic is used to quantify error associated with the measurements of people in a sample. Higher values indicate greater reliability [40]. This reliability statistic is comparable to the Cronbach alpha [42]. Cronbach alpha: This statistic was computed in RUMM 2030 to measure internal reliability. Test re-test reliability (TRT): TRT was established by asking participants to complete LIMB-Q Kids 2 weeks after the initial assessment. Intraclass correlation coefficient of 0.70 was considered acceptable in participants whose leg condition was stable during the time interval [45]. Anyone who reported an important change in the function, appearance and how they feel about their leg or who completed the TRT outside of 7–14 days was excluded. ICC with a two-way random effects model was computed using the transformed Rasch scores.
Dependency	Residual correlations between pairs of items are examined to identify any that were greater than 0.20, as high residual correlations can inflate reliability. For residual correlations greater than 0.20, a subtest is performed to determine the impact on reliability [40].
Targeting (Item locations)	The items of a scale are meant to define a continuum. We examined item locations to determine whether they were evenly spread over a reasonable range that matched the range of the construct reported by the sample [39]. Scales were examined graphically (person- item threshold distribution) and statistically (proportion of the sampleto score outside the range of each scale’s measurement).
Differential item functioning (DIF)	DIF is used to determine if individuals in subgroups (i.e., country, sex, age) responded differently to items despite the same measured trait level. A random sample is chosen to create equal-sized subgroups, with a proviso that there had to be a minimum of 200 to provide 50 for 4 class intervals [40]. When DIF was identified, variables were split for the relevant items, with both original and split person locations correlated to examine the impact of DIF on scale scoring.
Construct validity	Construct validity is about how well a scale measures the concept it was designed to measure. To examine construct validity, we transformed the Rasch logit scores into 0 (worse) to 100 (best) to test specific predefined hypothesis. P-values less than 0.05 were considered significant. Normality was assessed using Kurtosis(absolute > 2) and Skewness (absolute > 2), [46], and non-parametric statistics were applied if distributions were non-normal. We tested 16 hypotheses for this study (Table 5).

In addition to RMT analysis, Classical test theory (CTT) analysis was used to provide supplementary information about scale psychometric performance. CTT analysis included Test re-test (TRT) reliability and tests of construct validity (Table 1). For construct validity, if at least 75% of the predefined hypotheses are supported by the data (i.e., the results are in the expected direction or of the expected magnitude), a scale was considered to have adequate validity as per the COSMIN guidelines [43]. For the CTT analysis, Rasch logits were used to transform the scores from 0 to 100. For all scales, higher scores indicate a better outcome.

## Results

Eight hundred participants from 16 sites took part in the study. Table 2 provides sample characteristics. The mean age of the sample was 13.3 years (SD 3.2 years), with 45.5% of the sample as girls and 54.4 % boys. Based on the LLRS AIM index, 16% of participants scored as normal complexity, 66% as minimal complexity, 14.4% as moderate complexity and 3.6% as high complexity.

**Table 2:** Participant characteristics

Characteristic		N (800)	%
Age (years)	8 to 11	249	31.1
	12 to 14	276	34.5
	15 to 19	247	30.9
	20 to 25	24	3.0
Gender	Girl/Woman	361	45.4
	Boy/Man	433	54.4
	Other/ Not sure/ Prefer not to answer	2	0.3
Attend school	Yes	742	93.5
Country	Australia	37	4.6
	Canada	187	23.4
	Denmark	52	6.5
	England	209	26.1
	Germany	54	6.8
	India	50	6.3
	Scotland	37	4.6
	USA	174	21.8
LLRS AIM index score	Normal	128	16.0
	Minimal complexity	528	66.0
	Moderate complexity	115	14.4
	High/Substantial complexity	29	3.6
Diagnosis*	Leg Length Discrepancy	348	43.5
	Genu Valgum	145	18.1
	Fibular Hemimelia	99	12.4
	Skeletal Dysplasia	56	7.0
	Hemi-hypertrophy	45	5.6
	Congenital Femoral Deficiency	44	5.5
	Genu Varum	32	4.0
	Blount’s disease	29	3.6
	Idiopathic Leg Length Discrepancy	20	2.5
	Posteromedial Tibial Bowing	19	2.4
	Leg Length Discrepancy secondary to Perthes	17	2.1
	Leg Length Discrepancy secondary to Clubfoot	16	2.0
	Osteogenesis Imperfecta	14	1.8
	Congenital Pseudarthrosis of Tibia	10	1.3
	Patellar Instability	10	1.3
	Tibial Hemimelia	9	1.1
	Amputations (Due to trauma, infection, or other diagnosis)	6	0.8
	Post Traumatic Deformity	6	0.8
	Genu Recurvatum	5	0.6
	Perthes	4	0.5
	Hemi-atrophy	3	0.4
	Osteofibrous Dysplasia	3	0.4
	Trauma	3	0.4
	Achondroplasia	2	0.3
	Arthrogryposis	2	0.3
	Fibrous Dysplasia	2	0.3
	Growth Arrest	2	0.3
	Hereditary Multiple Exostosis	2	0.3
	Leg Length Discrepancy Secondary to Developmental Dysplasia Hip	2	0.3
	Neurofibromatosis Type 1	2	0.3
	Spina Bifida	2	0.3
	Other	48	6.0

*More than 1 can be reported

**Psychometric Findings** (Table 3 and Supplementary Tables 1–3)

**Table 3:** RMT scale level statistics and other scale findings

Scale	Items in Rasch analysis	Final Items N	%Scale Missing data	Sample N	RMT N	Score on scale %	Chi-square	DF	p-value	PSI	PSI	α + extr	α -extr	>0.30	FK Mean (Range)
										+ extr	-extr				
Leg Appearance	16	10	3.0	785	615	78.30	117.41	90	0.03	0.88	0.87	0.95	0.90	0	0.7 (0–3.6)
Physical Function	32 (25 DT)	11	2.0	799	588	73.60	133.35	99	0.01	0.84	0.86	0.93	0.90	1	0.1 (0–0.5)
Leg-related Distress	14 (14 DT)	11	2.0	794	622	78.30	107.02	77	0.01	0.76	0.78	0.90	0.86	1	2.6 (0–4.9)
Psychological Function	10	9	2.0	789	470	59.60	57.59	45	0.10	0.82	0.88	0.95	0.91	0	2.2 (0.5–5.2)
Social Function	12	10	3.0	790	550	69.60	75.21	60	0.09	0.76	0.80	0.91	0.86	0	1.6 (0–3.7)
Hip Symptoms	10	10	1.0	110	93		25.8	20	0.17	0.81	0.78	0.88	0.82	2	0.30 (0–1.0)
Leg Symptoms	14 (7 DT)	8	2.0	799	702		91.93	64	0.01	0.80	0.77	0.86	0.81	0	0.2 (0–0.8)
Knee Symptoms	13 (6 DT)	9	2.0	793	538		54.61	36	0.02	0.68	0.70	0.87	0.79	1	0.8 (0–2.4)
Ankle/Foot Symptoms	19	10	6.0	767	517		97.72	70	0.02	0.72	0.75	0.89	0.83	0	1.3 (0–2.8)

DF – Degrees of freedom; PSI = Person Separation Index; α = Chronbach alpha; FK = Flesch-Kincaid grade reading level

In the RMT analysis, one scale (School Function) was dropped due to low reliability (less than 0.70) and items from 2 scales were merged (Foot and Ankle Symptoms). In addition, to address disordered thresholds, we rescored the response options for 7 scales (i.e., the 2 middle response options were combined). After these adjustments, the RMT analysis provided evidence of reliability and validity for the following 8 scales: Physical Function, Leg Appearance, Leg Symptoms, Knee Symptoms, Foot and Ankle Symptoms, Leg-related Distress, Social Function, and Psychological Function. For the Hip Symptoms scale, to improve targeting, the RMT analysis was performed on the subset of participants who were identified by clinicians as having hip-related symptoms.

**Fig. 1:**
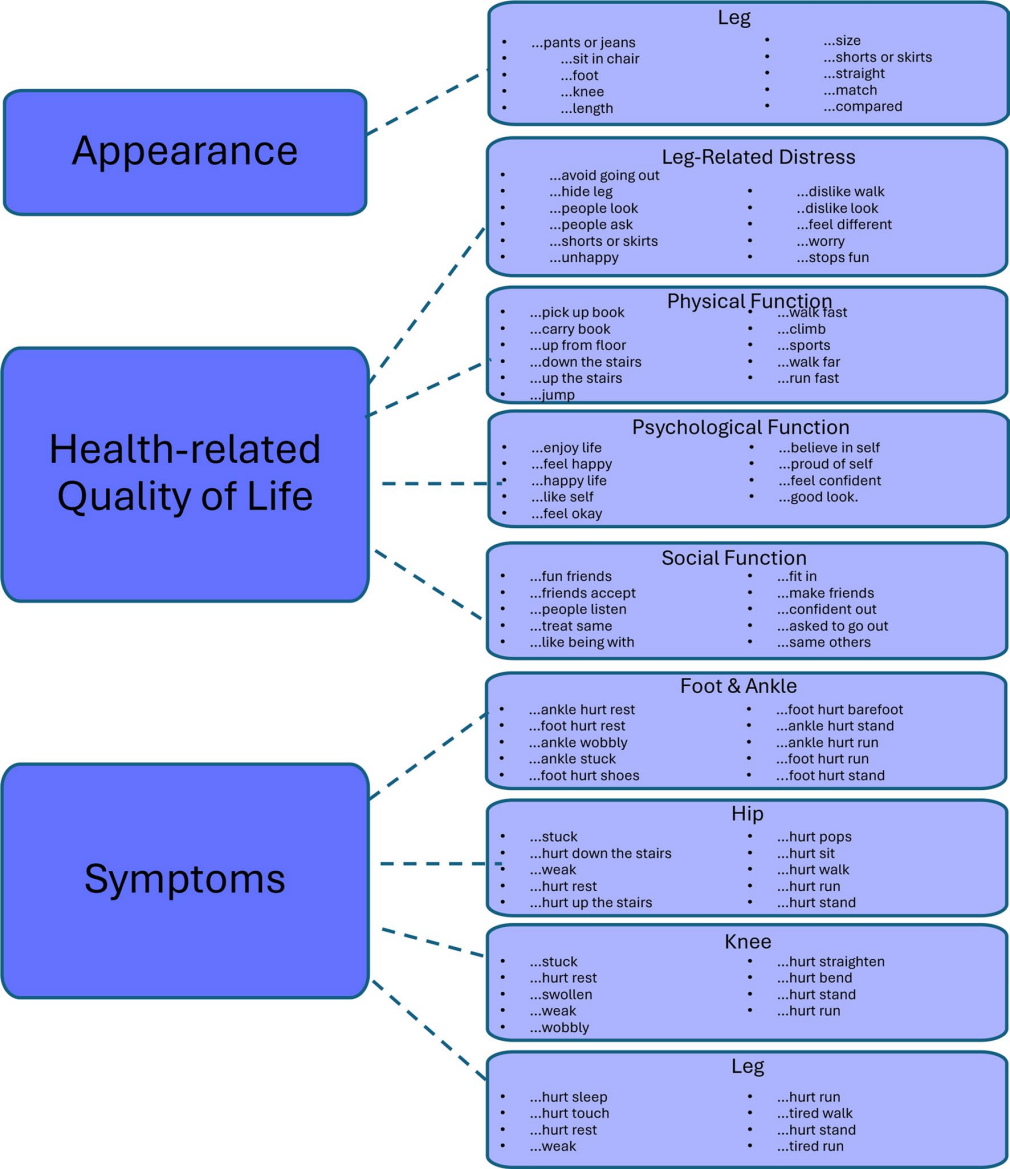
Mapping of LIMB-Q Kids conceptual framework to scales

RMT analysis led to LIMB-Q Kids being reduced from 159 to 88 items. Final conceptual framework and mapping to individual scales and items is presented in Fig. 1. Supplementary Table 1 shows the item fit statistics for each scale. All items had ordered thresholds. Item fit to the Rasch model was good, as all items had non-significant chi-square p-values after Bonferroni adjustment and fit residuals for 75 items were within ± 2.5. Supplementary Table 1 shows findings for differential item functioning (DIF), which was evident for 1 item by country, 2 items by age group, and 2 items for gender. For the items where DIF was found, split item analysis was performed, and Pearson correlations between the original person locations and person locations after split analysis were ≥ 0.999 showing little impact on scoring. Hence these items were retained as it is.

Table 3 shows the scale-level results. The reliability in terms of Person Separation Index (PSI) value for one scale with extremes included fell slightly below 0.70. All other PSI values and all Cronbach alpha values were above 0.70 except the Knee Symptoms scale. In terms of local dependency, 1 pair of items in each of 3 scales (Physical Function, Leg-related Distress, Knee Symptoms) and 2 pairs of items in 1 scale (Hips Symptoms) had residual correlations > 0.30. Subtests performed between the pairs of items reduced the reliability statistics by a maximum of 0.06 for the Hip Symptoms, 0.03 for Knee Symptoms, and 0.02 for Physical Function and Leg-related Distress. The reliability statistics after subtests were performed remained above 0.70 for all scales except the Knee Symptoms scale, where the PSI values were 0.65 and 0.67 with and without extremes included, with Cronbach alpha values 0.85 and 0.76 with and without extremes. Floor effects for all scales were less than 1%. Ceiling effects range from 5% (Leg Symptoms scale) to 40.2% {(Psychological Function scale) Supplementary Table 2a}. After excluding participants who were rated normal or minimal complexity as per the LLRS AIM index from the sample, the ceiling effects for all limb-specific scales was under 15 % (Supplementary Table 2b).

Table 4a shows that the ICC values based on a sample of 46 participants who completed LIMB-Q Kids twice, with in 7–14 days, ranged from 0.77–0.95. Table 4b shows the ICC values based on a sample of 75 participants who completed LIMB-Q Kids twice, with in 5–35 days, ranged from 0.66–0.92.

**Table 4a:** Test re-test reliability

LIMB-Q Kids Scale	N**	ICC*	Lower	Upper
Leg Appearance	42	0.77	0.57	0.87
Physical Function	38	0.92	0.85	0.96
Leg-related Distress	46	0.94	0.89	0.97
Psychological Function	45	0.93	0.88	0.96
Social Function	45	0.92	0.85	0.95
Hip Symptoms	46	0.95	0.91	0.97
Leg Symptoms	46	0.91	0.83	0.95
Knee Symptoms	46	0.92	0.85	0.95
Foot and Ankle Symptoms	46	0.84	0.70	0.91

*ICC Intraclass correlation coefficient

**Participants who completed second LIMB-Q Kids within 7–14 days

**Table 4b:** Test re-test reliability

LIMB-Q Kids Scale	N**	ICC*	Lower	Upper
Leg Appearance	75	0.66	0.45	0.78
Physical Function	75	0.86	0.77	0.91
Leg-related Distress	75	0.92	0.88	0.95
Psychological Function	75	0.90	0.84	0.94
Social Function	75	0.90	0.83	0.93
Hip Symptoms	75	0.92	0.88	0.95
Leg Symptoms	75	0.89	0.82	0.93
Knee Symptoms	75	0.90	0.85	0.94
Foot and Ankle Symptoms	75	0.88	0.81	0.93

*ICC Intraclass correlation coefficient

**Participants who completed second LIMB-Q Kids within 5–35 days (Mean 15 days, SD 5.4 days). None of the participants reported change in any of the domains between the first and second administration of LIMB-Q Kids

### Construct validity

Table 5 and Supplementary Tables 4–16 show the construct validation results. Overall, 15 of 16 (93.75%) hypotheses provided evidence for construct validity (>75% hypotheses needed to be met). For individual scales, all stated hypotheses were met with the exception for the Foot and Ankle Symptom scale where 3 of 4 hypotheses were met.

**Fig. 2:**
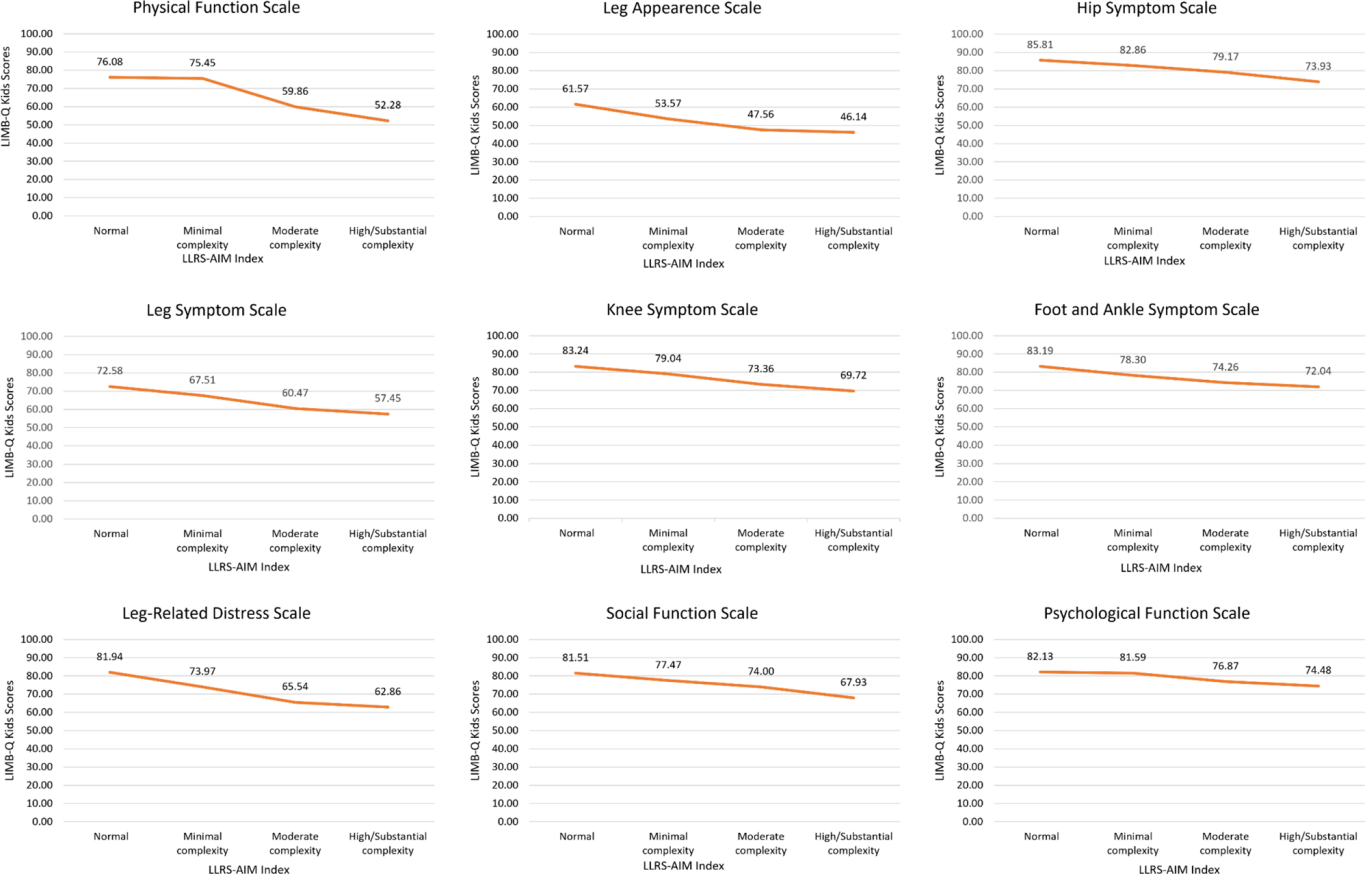
LIMB-Q kids scale scores decrease with increasing severity based on LLRS AIM index

Girls in our sample scored lower than boys on 7 LIMB-Q Kids scales. No statistically significant difference by gender was found for the Leg Appearance scale and the Social Function scale (Table 6). Analysis by age (Table 7) showed that LIMB-Q Kids scores were not correlated with age (all r less than 0.052). Finally, scores on all LIMB-Q Kids scales were incrementally lower with increasing severity based on the LLRS AIMs index (Fig. 2).

**Table 5:** Construct validity hypotheses

Hypothesis statement	Physical Function	Leg Appearance	Hip Symptom	Leg Symptom	Knee Symptom	Foot and Ankle Symptom	Leg-related Distress	Social Function	Psychological Function
1. LIMB-Q Kids Physical Function scale correlates with PEDSQL related > 0.5	**Y**	NA	NA	NA	NA	NA	NA	NA	NA
2. LIMB-Q Kids Physical Function scale correlates with PROMIS Mobility related > 0.5	**Y**	NA	NA	NA	NA	NA	NA	NA	NA
3. LIMB-Q Kids Social Function scale correlates with PEDSQL Social Scale related > 0.5	NA	NA	NA	NA	NA	NA	NA	**Y**	NA
4. LIMB-Q Kids Social Function scale correlates with PEDSQL Emotion Scale related > 0.5	NA	NA	NA	NA	NA	NA	NA	NA	**Y**
5. LIMB-Q Kids Leg-Appearance scale should be not related to LIMB-Q Kids symptom scales < 0.3	NA	NA	**Y**	**Y**	**Y**	**Y**	NA	NA	NA
6. LIMB-Q Kids Leg-Appearance and Leg-related Distress scales 0.3–0.5 related but dissimilar	NA	**Y**	NA	NA	NA	NA	**Y**	NA	NA
7. LIMB-Q Kids Leg-related Distress scales correlate with LIMB-Q Kids Psychological Function scale > 0.5	NA	NA	NA	NA	NA	NA	**Y**	NA	**Y**
8. LIMB-Q Kids Leg-related Distress scale correlates with LIMB-Q Kids Social Function scale 0.3–0.5	NA	NA	NA	NA	NA	NA	**Y**	**Y**	NA
9. LIMB-Q Kids Leg-Appearance, Social Function, and Psychological Function scales will correlate 0.3–0.5	NA	**Y**	NA	NA	NA	NA	NA	**Y**	**Y**
10. LIMB-Q Kids Physical Function and Leg-related Distress scales are related but dissimilar 0.3-05	**Y**	NA	NA	NA	NA	NA	**Y**	NA	NA
11. With increased severity based on the LLRS-AIM index, LIMB-Q Kids scale scores will decrease	**Y**	**Y**	**Y**	**Y**	**Y**	**Y**	**Y**	**Y**	**Y**
12. If the clinical team reported symptoms for a body part score on the corresponding symptom scale will be lower	NA	NA	**Y**	**Y**	**Y**	**Y**	NA	NA	NA
13. With the increase in the number of deformities on the LLRS AIM index, LIMB-Q Kids scale scores decrease	**Y**	**Y**	**Y**	**Y**	**Y**	**N**	**Y**	NA	NA
14. Patients in frame report lower scores on LIMB-Q Kids scales as compared to patients who are > 6 months post-frame removal	**Y**	**Y**	NA	NA	NA	NA	NA	NA	NA
15. For participants reporting liking their scars, LIMB-Q Kids scale scores increase with increase in the likeness	NA	**Y**	NA	NA	NA	NA	**Y**	**Y**	**Y**
16. For participants reporting liking the appearance of their leg, LIMB-Q Kids scale scores increase with the increase in the likeness	NA	**Y**	NA	NA	NA	NA	**Y**	NA	NA
Proportion of the Hypothesis met	**6/6**	**7/7**	**4/4**	**4/4**	**4/4**	**3/4**	**8/8**	**5/5**	**5/5**

Note: Y, yes hypothesis met and p-value was significant; N, no hypothesis rejected, NA, not applicable

**Table 6:** Analysis by gender

	Gender	N	Mean Score	Std. Deviation	Std. Error Mean	Mean diff	p-value
Physical Function	Girl	361	68.60	24.09	1.27	−7.2	<0.001
	Boy	432	75.78	23.24	1.12		
Leg Appearance	Girl	355	51.29	25.42	1.35	−4.4	0.026
	Boy	423	55.66	29.24	1.42		
Hip Symptoms	Girl	361	78.84	19.97	1.05	−6.74	<0.001
	Boy	430	85.58	18.41	0.89		
Leg Symptoms	Girl	361	63.73	19.64	1.03	−5.9	<0.001
	Boy	432	69.69	19.56	0.94		
Knee Symptoms	Girl	360	74.06	20.12	1.06	−8.2	<0.001
	Boy	426	82.24	18.13	0.88		
Foot and Ankle Symptoms	Girl	350	75.60	20.43	1.09	−5	<0.001
	Boy	411	80.55	19.34	0.95		
Leg-related Distress	Girl	359	68.87	21.43	1.13	−8.6	<0.001
	Boy	429	77.52	18.63	0.90		
Social Function	Girl	355	75.35	20.01	1.06	−3.5	0.013
	Boy	429	78.89	19.56	0.94		
Psychological Function	Girl	355	77.92	21.49	1.14	−5.2	<0.001
	Boy	428	83.16	19.86	0.96

**Table 7:** Analysis by age

		Physical Function	Leg Appearance	Hip Symptoms	Leg Symptoms	Knee Symptoms	Foot and Ankle Symptoms	Leg-Related Distress	Social Function	Psychological Function
Age years	Pearson Correlation	−.073*	0.052	−.131**	−.076*	−.082*	−0.005	−.095**	−0.002	−.171**
	Sig. (2-tailed)	0.04	0.146	<.001	0.032	0.022	0.897	0.007	0.949	<.001
	N	795	780	793	795	788	763	790	786	785

**Correlation is significant at the 0.01 level (2-tailed)

*Correlation is significant at the 0.05 level (2-tailed)

## Discussion

This international field test study provided evidence for the reliability and validity of 9 LIMB-Q Kids scales. The DIF analysis to explore DIF by age, gender, and country provided evidence to support a common scoring algorithm for each LIMB-Q Kids scale. The School scale had PSI values below 0.70 in the analysis and a decision was made to drop this scale from the module. The final version of LIMB-Q Kids fills an important gap in the literature by providing an internationally applicable PROM for use in children and adolescents with LLDs. This PROM was developed using rigorous methods that followed international guidelines for PROM development and validation. We engaged patients with LLDs at every step of development (including qualitative interviews for concept elicitation and content validation study and opportunity to provide feedback during the field test study), and our findings meet the criteria listed on the COSMIN checklist for assessing the methodological quality of studies on measurement properties of health status instruments [17, 18, 28, 44]. This PROM has a total of 88 items spread over 9 independently functioning scales. It is estimated that it will take approximately 10 minutes to complete all 9 scales. However, the modular design provides the flexibility to have patients only complete scales as appropriate for clinical care or research needs at a given time point.

Before developing the LIMB-Q Kids, our systematic review indicated the lack of a PROM developed and validated specifically for children with LLDs [15]. Since the publication of our systematic review in 2017, other lower limb-specific instruments have been published, such as ad hoc limb deficiencies questionnaire [45], Limb Lengthening Satisfaction Questionnaire (LLSQ), Childhood Amputee Prosthetics Project-Prosthetics Satisfaction Inventory (CAPP-PSI) [13, 45, 46], the Limb-Deformity SRS questionnaire (LD-SRS) [47, 48], and Gait Outcomes Assessment List—-Lower Limb Differences (GOAL-LD) [49, 50]. Limitations associated with these PROMs include that they were either not developed and validated specifically for children, were developed for other orthopaedic conditions and later adapted for LLDs or were developed without any direct input from children with LLDs. The LD-SRS questionnaire, an adaptation of the Scoliosis Research Society (SRS) questionnaire, was recently used in study of 30 pediatric patients with lower limb deformities [48]. This PROM was developed for patients aged 18 years or older, with spine deformities and was modified by replacing the word “back” or “trunk” with “limb” to form the LD-SRS [47]. GOAL-LD was developed for children 9–18 years with LLDs for their gait outcome assessment by modifying the GOAL for children with cerebral palsy [50]. The GOAL-LD assesses domains such as walking and getting around, pain, discomfort, fatigue, physical activities, games, and recreation, gait appearance, use of braces and assistive devices, and body image and self-esteem. While the initial validation studies for GOAL-LD in a sample of 137 children are promising, further evidence of validity and reliability from a larger diverse sample of patients from different cultures and countries (languages) is needed [49].

### Clinical applications

LIMB-Q Kids can provide insights into how limb conditions affect a child’s body image and physical, psychological, and social well-being providing a holistic assessment. This new PROM can also help identify areas of concern (e.g., leg-related distress, appearance-related concerns) to guide individualized treatment plans and support. LIMB-Q Kids can be used to measure patient-reported outcomes for complex life-altering surgeries, such as limb reconstruction or amputation. LIMB-Q Kids can also be used by clinics or health systems to evaluate the effectiveness of interventions and improve care delivery for patients with limb differences once future longitudinal research provides evidence for the responsiveness of this new PROM. LIMB-Q Kids can provide a structured way to incorporate the child’s perspective into clinical conversations, fostering shared decision-making and enhancing patient engagement.

### Limitations

Patients with isolated hip, knee, and foot disorders were not included in the qualitative sample or field-test study since they are considered clinically different populations. Research is needed to determine if LIMB-Q Kids measures outcomes that matter to children with isolated hip, knee, and foot disorders, or whether a new PROM is needed. Recruitment took place at pediatric orthopaedic centers that have different criteria for the age at which patients are transferred to adult orthopaedic centres. As such, a small number of patients aged 20–25 years were still been seen by the pediatric orthopaedic centres included in this study and hence were included in the sample. The sample used in the RMT analysis for the Hip Symptom scale was small (N = 110) and the results should be treated with caution. One scale had lower PSI values that dropped below 0.70 when local dependency was taken into account. However, the Cronbach alpha and TRT values for this scale were all above 0.70. The sample size for the TRT was below the sample required for an “adequate” rating in terms of COSMIN criteria.

In our study sample, ceiling effects of more than 15% were seen in some LIMB-Q Kids scales (Physical Function, Leg-related Distress, Social Function, and Psychological Function). We attribute the ceiling effects seen in some of the LIMB-Q Kids scales to the fact that we did not have a true ‘no treatment group’ in our sample. Patients had already received some treatment for their limb differences at the time of completion of LIMB-Q Kids. Moreover, the leg condition for most of the sample was rated normal to minimal complexity as per the LLRS AIM index while only 18% were moderate to high complexity. Future research should include participants with higher complexity and those who have not received treatment for their limb differences at the time of competition of LIMB-Q Kids to determine whether the ceiling effect observed in some scales during the field test study are true. If they are truly present, one of the ways to address this issue includes adding additional items or subscales that assess higher levels of performance or complex skills.

### Conclusions and next steps

This large international field test study provides evidence for the validity and reliability of LIMB-Q Kids. Validation of a PROM is an ongoing process that involves examining a range of measurement properties over time. The study we performed was cross-sectional. Future longitudinal research is needed to evaluate the responsiveness (ability to detect change overtime) of LIMB-Q Kids and establish minimal clinically important differences (MCID). The results from these studies can aid in score interpretation for clinical and research purposes. To advance the field and facilitate evidence-based decision-making, a rigorously designed LLD-specific PROM is needed. LIMB-Q Kids can be used in research and in clinical care to measure outcomes that matter to patients, such as physical, social, psychological function, and leg-related distress, and appearance-related concerns. These data could supplement other objective outcome information to facilitate shared decision-making and help families decide on their child’s treatment [51]. LIMB-Q Kids is now available in English, Hindi, Danish, and German and several other translations are now in progress. LIMB-Q Kids is available for use in clinical care and research and can be obtained at http://www.qportfolio.org. Further information about LIMB-Q Kids can be obtained at https://www.limbnetwork.com/limbq-kids.

## Electronic supplementary material

Below is the link to the electronic supplementary material.

Supplementary Material 1

## Data Availability

The dataset analyzed during the current study may be available from the corresponding author upon reasonable request and contingent upon approval from the ethics aboard.

## Abbreviations

PROM: Patient-Reported Outcome Measure
LLD: Lower Limb Difference
PROMIS: Patient-Reported Outcomes Measurement Information System
PedsQL: Pediatric Quality of Life Inventory
LLRS-AIM: Limb Lengthening and Reconstruction Society – AIM Index
RMT: Rasch Measurement Theory
HRQL: Health-Related Quality of Life
REDCap: Research Electronic Data Capture
COSMIN: Consensus-based Standards for the Selection of Health Measurements Instruments
CTT: Classical Test Theory
TRT: Test-Retest
PSI: Person Separation Index
DIF: Differential Item Functioning
CAPP-PSI: Childhood Amputee Prosthetics Project - Prosthetics Satisfaction Inventory
LD-SRS: Limb Deformity - Scoliosis Research Society Questionnaire
GOAL-LD: Gait Outcomes Assessment – Lower Limb Differences
SRS: Scoliosis Research Society
