# Supplementary material for: Psychometric findings for LIMB-Q kids based on an international study of 800 children and adolescents with lower limb differences
Source: J Patient Rep Outcomes. 2025 Jul 5;9:82. doi: 10.1186/s41687-025-00916-y (PMC12228855; doi:10.1186/s41687-025-00916-y)
Supplement: Supplementary file 1 — Supplementary Material 1 [file 41687_2025_916_MOESM1_ESM.docx]

**Supplementary Table 1: RMT item level fit statistics and Differential Item Functioning results**

| Scales | Item Fit Statistics | | | | | | | DIF | | |
| --- | --- | --- | --- | --- | --- | --- | --- | --- | --- | --- |
| Item | Location | SE | Fit  Residual | DF | c^2^ | DF | p-value | Age | Gender | Country |
| LEG APPEARANCE | | | | | | | | | | |
| 1. …pants or jeans | -0.41 | 0.05 | 0.42 | 550 | 13.56 | 9.00 | 0.14 | - | - | - |
| 1. …sit in chair | -0.39 | 0.05 | -2.09 | 549 | 14.33 | 9.00 | 0.11 | - | - | - |
| 1. …foot | -0.07 | 0.05 | 3.62 | 547 | 11.27 | 9.00 | 0.26 | - | - | - |
| 1. …knee | -0.03 | 0.05 | -0.90 | 550 | 8.00 | 9.00 | 0.53 | - | - | - |
| 1. …length | -0.01 | 0.05 | 3.42 | 550 | 9.24 | 9.00 | 0.42 | - | - | - |
| 1. …size | 0.02 | 0.05 | -1.74 | 550 | 10.38 | 9.00 | 0.32 | - | - | - |
| 1. …shorts or skirts | 0.08 | 0.05 | -1.85 | 550 | 15.18 | 9.00 | 0.09 | - | - | - |
| 1. …straight | 0.12 | 0.05 | -0.51 | 550 | 5.36 | 9.00 | 0.80 | - | - | - |
| 1. …match | 0.34 | 0.05 | 3.75 | 550 | 13.33 | 9.00 | 0.15 | - | - | - |
| 1. …compared | 0.37 | 0.05 | -1.36 | 549 | 16.76 | 9.00 | 0.05 | - | - | - |
| PHYSICAL FUNCTION | | | | | | | | | | |
| 1. …pick up book | -2.46 | 0.12 | 1.32 | 531 | 13.49 | 9.00 | 0.14 | - | - | - |
| 1. …carry book | -1.05 | 0.10 | -2.70 | 532 | 9.60 | 9.00 | 0.38 | - | - | - |
| 1. …up from floor | -0.85 | 0.09 | 3.51 | 530 | 18.14 | 9.00 | 0.03 | - | - | - |
| 1. …down the stairs | -0.84 | 0.09 | -1.07 | 532 | 6.91 | 9.00 | 0.65 | - | - | - |
| 1. …up the stairs | -0.69 | 0.09 | -1.20 | 533 | 7.57 | 9.00 | 0.58 | - | - | - |
| 1. …jump | 0.35 | 0.08 | -1.35 | 532 | 12.31 | 9.00 | 0.20 | - | - | Yes |
| 1. …walk fast | 0.47 | 0.08 | -1.65 | 533 | 17.28 | 9.00 | 0.04 | - | - | - |
| 1. …climb | 0.86 | 0.08 | -1.88 | 532 | 14.77 | 9.00 | 0.10 | - | - | - |
| 1. …sports | 1.15 | 0.08 | 0.80 | 529 | 7.35 | 9.00 | 0.60 | - | - | - |
| 1. …walk far | 1.18 | 0.08 | 0.39 | 533 | 1.94 | 9.00 | 0.99 | - | - | - |
| 1. …run fast | 1.89 | 0.08 | -2.90 | 532 | 24.00 | 9.00 | 0.00 | - | - | - |
| LEG SYMPTOMS | | | | | | | | | | |
| 1. …hurt sleep | -2.04 | 0.09 | 2.34 | 612 | 10.58 | 8.00 | 0.23 | - | - | - |
| 1. …hurt touch | -1.51 | 0.09 | 1.59 | 610 | 12.39 | 8.00 | 0.13 | - | - | - |
| 1. …hurt rest | -1.26 | 0.09 | 0.21 | 612 | 9.19 | 8.00 | 0.33 | - | - | - |
| 1. …weak | -0.12 | 0.08 | 2.44 | 610 | 8.28 | 8.00 | 0.41 | - | - | - |
| 1. …hurt run | 0.92 | 0.07 | -3.62 | 608 | 16.88 | 8.00 | 0.03 | - | - | - |
| 1. …tired walk | 1.03 | 0.07 | -3.89 | 610 | 15.49 | 8.00 | 0.05 | - | - | - |
| 1. …hurt stand | 1.15 | 0.08 | -0.42 | 612 | 5.71 | 8.00 | 0.68 | - | - | - |
| 1. …tired run | 1.83 | 0.07 | -3.91 | 608 | 13.40 | 8.00 | 0.10 | - | - | - |
| KNEE SYMPTOMS | | | | | | | | | | |
| 1. …stuck | -1.01 | 0.11 | -0.11 | 475 | 3.46 | 4.00 | 0.48 | - | - | - |
| 1. …hurt rest | -0.80 | 0.10 | -0.59 | 476 | 5.00 | 4.00 | 0.29 | - | - | - |
| 1. …swollen | -0.62 | 0.10 | -0.74 | 476 | 3.68 | 4.00 | 0.45 | - | - | - |
| 1. …weak | -0.24 | 0.09 | -1.43 | 476 | 11.94 | 4.00 | 0.02 | - | - | - |
| 1. …wobbly | -0.23 | 0.09 | -1.12 | 475 | 6.70 | 4.00 | 0.15 | - | - | - |
| 1. …hurt straighten | 0.11 | 0.09 | -1.08 | 474 | 6.83 | 4.00 | 0.15 | - | - | - |
| 1. …hurt bend | 0.21 | 0.09 | -1.01 | 473 | 7.35 | 4.00 | 0.12 | - | - | - |
| 1. …hurt stand | 1.23 | 0.08 | 0.08 | 475 | 3.95 | 4.00 | 0.41 | - | - | - |
| 1. …hurt run | 1.35 | 0.08 | 0.92 | 469 | 5.70 | 4.00 | 0.22 | - | - | - |
| FOOT & ANKLE SYMPTOMS | | | | | | | | | | |
| 1. …ankle hurt rest | -1.36 | 0.11 | -1.53 | 463 | 10.10 | 7.00 | 0.18 | - | - | - |
| 1. …foot hurt rest | -1.31 | 0.11 | 0.81 | 459 | 5.83 | 7.00 | 0.56 | - | - | - |
| 1. …ankle wobbly | -0.97 | 0.10 | 0.19 | 462 | 9.63 | 7.00 | 0.21 | - | - | - |
| 1. …ankle stuck | -0.73 | 0.10 | 0.63 | 462 | 9.73 | 7.00 | 0.20 | - | - | - |
| 1. …foot hurt shoes | -0.08 | 0.09 | -1.48 | 459 | 13.38 | 7.00 | 0.06 | - | - | - |
| 1. …foot hurt barefoot | 0.51 | 0.08 | 0.18 | 459 | 4.28 | 7.00 | 0.75 | - | - | - |
| 1. …ankle hurt stand | 0.69 | 0.08 | -1.17 | 463 | 12.62 | 7.00 | 0.08 | - | - | - |
| 1. …ankle hurt run | 0.85 | 0.08 | 0.07 | 459 | 11.84 | 7.00 | 0.11 | - | - | - |
| 1. …foot hurt run | 1.13 | 0.08 | -2.89 | 456 | 11.42 | 7.00 | 0.12 | - | - | - |
| 1. …foot hurt stand | 1.27 | 0.09 | -0.51 | 459 | 8.91 | 7.00 | 0.26 | - | - | - |
| HIP SYMPTOMS | | | | | | | | | | |
| 1. …stuck | -1.54 | 0.25 | 2.11 | 81.76 | 5.23 | 2.00 | 0.07 | NA | NA | NA |
| 1. …hurt down the stairs | -0.72 | 0.22 | -1.20 | 80.88 | 3.73 | 2.00 | 0.15 | NA | NA | NA |
| 1. …weak | -0.59 | 0.21 | 1.07 | 81.76 | 0.27 | 2.00 | 0.87 | NA | NA | NA |
| 1. …hurt rest | -0.50 | 0.22 | 0.05 | 80.88 | 0.79 | 2.00 | 0.67 | NA | NA | NA |
| 1. …hurt up the stairs | -0.34 | 0.21 | -1.97 | 81.76 | 8.07 | 2.00 | 0.02 | NA | NA | NA |
| 1. …hurt pops | 0.15 | 0.18 | 0.76 | 81.76 | 0.84 | 2.00 | 0.66 | NA | NA | NA |
| 1. …hurt sit | 0.18 | 0.20 | -0.06 | 81.76 | 1.80 | 2.00 | 0.41 | NA | NA | NA |
| 1. …hurt walk | 0.55 | 0.20 | -0.70 | 80.88 | 2.52 | 2.00 | 0.28 | NA | NA | NA |
| 1. …hurt run | 1.23 | 0.17 | -0.46 | 81.76 | 1.57 | 2.00 | 0.46 | NA | NA | NA |
| 1. …hurt stand | 1.57 | 0.21 | -0.57 | 81.76 | 1.01 | 2.00 | 0.60 | NA | NA | NA |
| LEG-RELATED DISTRESS | | | | | | | | | | |
| 1. …avoid going out | -1.66 | 0.11 | -1.15 | 563 | 10.66 | 7.00 | 0.15 | - | - | - |
| 1. …hide leg | -0.47 | 0.08 | -0.42 | 563 | 6.43 | 7.00 | 0.49 | - | - | - |
| 1. …people look | -0.38 | 0.08 | -1.59 | 563 | 13.95 | 7.00 | 0.05 | - | Yes | - |
| 1. …people ask | -0.26 | 0.08 | 1.09 | 563 | 11.22 | 7.00 | 0.13 | - | - | - |
| 1. …shorts or skirts | -0.11 | 0.08 | 0.73 | 563 | 4.32 | 7.00 | 0.74 | - | - | - |
| 1. …unhappy | 0.18 | 0.08 | -3.37 | 563 | 18.17 | 7.00 | 0.01 | - | - | - |
| 1. …dislike walk | 0.21 | 0.07 | -1.59 | 563 | 6.17 | 7.00 | 0.52 | - | - | - |
| 1. …dislike look | 0.32 | 0.07 | -2.47 | 563 | 13.96 | 7.00 | 0.05 | - | - | - |
| 1. …feel different | 0.53 | 0.07 | -1.63 | 563 | 7.81 | 7.00 | 0.35 | - | - | - |
| 1. …worry | 0.72 | 0.08 | 1.07 | 563 | 5.87 | 7.00 | 0.56 | - | - | - |
| 1. …stops fun | 0.93 | 0.07 | 1.70 | 562 | 8.48 | 7.00 | 0.29 | - | - | - |
| PSYCHOLOGICAL FUNCTION | | | | | | | | | | |
| 1. …enjoy life | -0.81 | 0.09 | -1.03 | 414 | 5.98 | 5.00 | 0.31 | - | - | - |
| 1. …feel happy | -0.66 | 0.09 | 0.14 | 414 | 3.57 | 5.00 | 0.61 | - | - | - |
| 1. …happy life | -0.56 | 0.09 | 1.20 | 414 | 1.80 | 5.00 | 0.88 | - | - | - |
| 1. …like self | -0.23 | 0.09 | -4.04 | 414 | 17.19 | 5.00 | 0.00 | - | - | - |
| 1. …feel okay | 0.09 | 0.08 | -2.00 | 414 | 8.61 | 5.00 | 0.13 | - | - | - |
| 1. …believe in self | 0.11 | 0.08 | -1.00 | 415 | 1.26 | 5.00 | 0.94 | - | - | - |
| 1. …proud of self | 0.30 | 0.08 | 0.71 | 414 | 5.78 | 5.00 | 0.33 | - | - | - |
| 1. …feel confident | 0.68 | 0.08 | -0.10 | 415 | 7.64 | 5.00 | 0.18 | - | - | - |
| 1. …good look | 1.07 | 0.08 | 1.20 | 415 | 5.76 | 5.00 | 0.33 | - | - | - |
| SOCIAL FUNCTION | | | | | | | | | | |
| 1. …fun friends | -1.52 | 0.09 | -1.42 | 492 | 8.10 | 6.00 | 0.23 | - | - | - |
| 1. …friends accept | -1.09 | 0.09 | -1.14 | 491 | 10.78 | 6.00 | 0.10 | - | - | - |
| 1. …people listen | -0.33 | 0.07 | 0.79 | 491 | 3.20 | 6.00 | 0.78 | Yes | - | - |
| 1. …treat same | -0.20 | 0.07 | -1.01 | 490 | 7.96 | 6.00 | 0.24 | - | - | - |
| 1. …like being with | -0.06 | 0.07 | 1.39 | 491 | 5.84 | 6.00 | 0.44 | - | - | - |
| 1. …fit in | 0.27 | 0.06 | -2.81 | 490 | 19.21 | 6.00 | 0.00 | - | - | - |
| 1. …make friends | 0.64 | 0.06 | 0.31 | 491 | 3.61 | 6.00 | 0.73 | - | - | - |
| 1. …confident out | 0.65 | 0.06 | 0.84 | 488 | 5.30 | 6.00 | 0.51 | Yes | - | - |
| 1. …asked to go out | 0.78 | 0.06 | 2.14 | 491 | 5.32 | 6.00 | 0.50 | - | 1 | - |
| 1. …same others | 0.85 | 0.06 | 0.00 | 488 | 5.89 | 6.00 | 0.44 | - | - | - |

SE = standard error; DF = degrees of freedom; Significant DIF: 1 = random sample 1, 2 = random sample 2, 3 = random sample 3

Pearson Correlations- Distress: Gender = 1.000; Social: Age 1, 2, 3 = 0.999; Social: Gender 1 = 1.000; Function: Country 3 = 0.99

**Supplementary Table 2 a) : Floor and Ceiling Effects**

|  | N | Floor % | Ceiling % |
| --- | --- | --- | --- |
| All participants | | | |
| Physical Function | 799 | 0.5 | 25.9 |
| Leg Appearance | 784 | 8.4 | 13.3 |
| Leg-related Distress | 794 | 0.3 | 21.4 |
| Social Function | 790 | 0.5 | 29.9 |
| Psychological Function | 789 | 0.3 | 40.2 |
| Participants where clinicians reported no clinical symptoms in these body parts | | | |
| Hip Symptoms | 110 | 0.9 | 14.5 |
| Knee Symptoms | 218 | 0.5 | 13.8 |
| Foot & Ankle Symptoms | 123 | 0.8 | 11.4 |
| Leg Symptoms | 302 | 0 | 5.0 |

**Supplementary Table 2 b) : Floor and Ceiling Effects**

|  | N* | Floor % | Ceiling % |
| --- | --- | --- | --- |
| All participants | | | |
| Physical Function | 144 | 2.1 | 6.9 |
| Leg Appearance | 142 | 9.0 | 5.6 |
| Leg-related Distress | 144 | 0.7 | 11.8 |
| Social Function | 143 | 0.7 | 21.5 |
| Psychological Function | 142 | 0.7 | 31.9 |

***All participants excluding Normal and Minimal Complexity patients based on LLRS AIM Index**

**Supplementary Table 3: Threshold maps and person-item threshold distributions**

| **LEG-APPEARANCE** | |
| --- | --- |
| 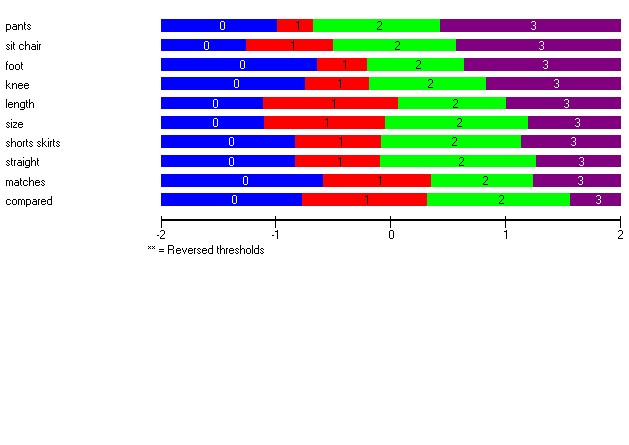 | 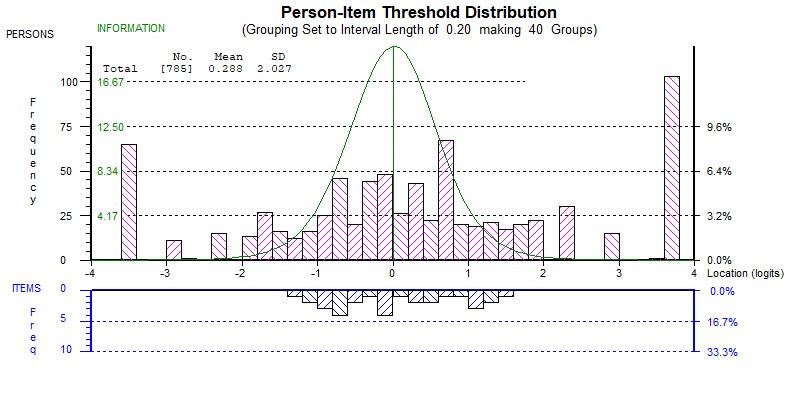 |
| **PHYSICAL FUNCTION** | |
| 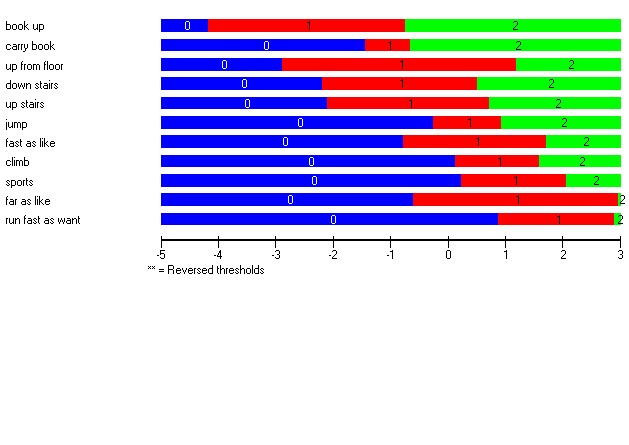 | 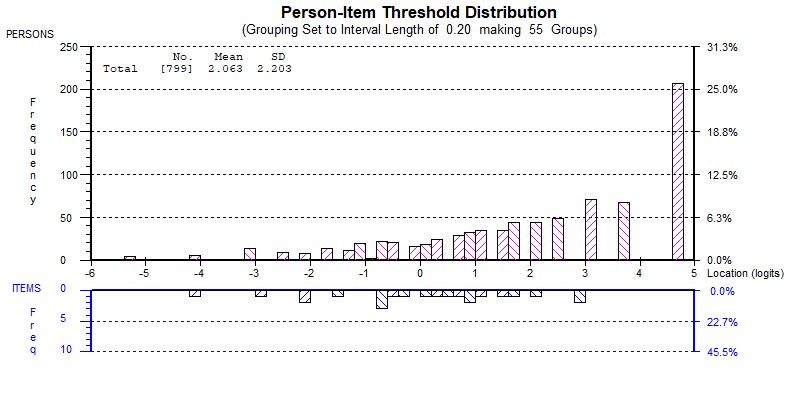 |
| **LEG SYMPTOMS** | |
| 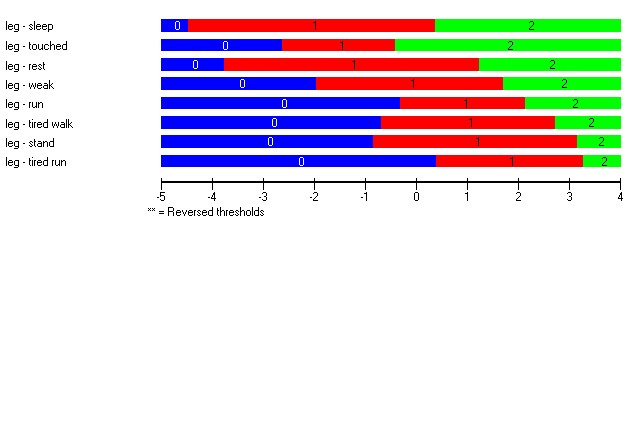 | 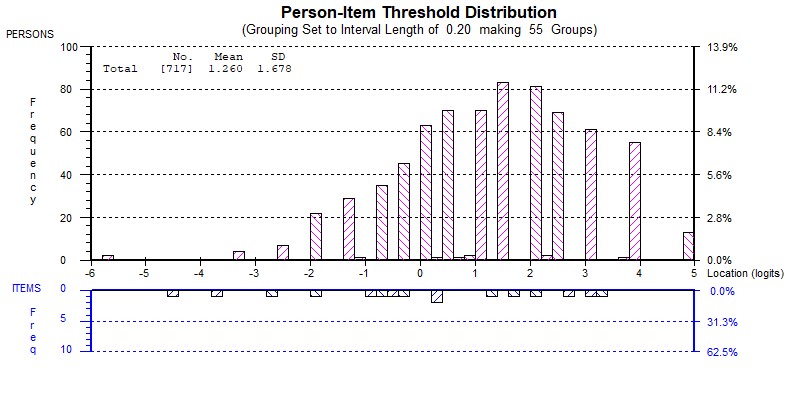 |
| **KNEE SYMPTOMS** | |
| 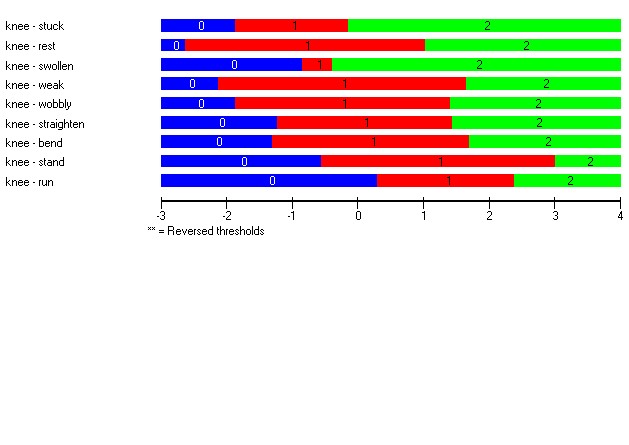 | 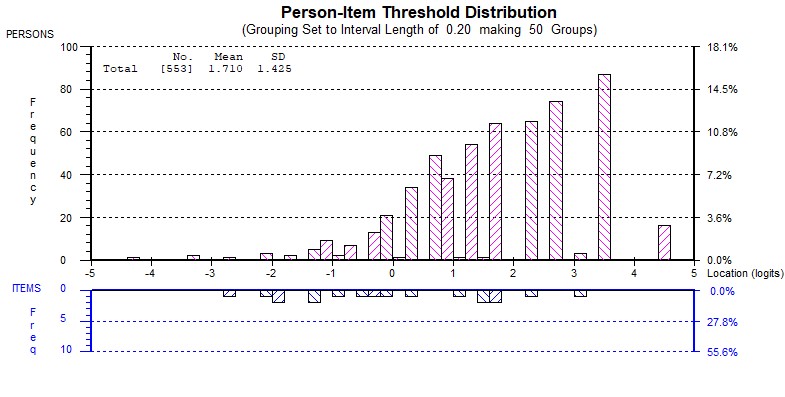 |
| **FOOT & ANKLE SYMPTOMS** | |
| 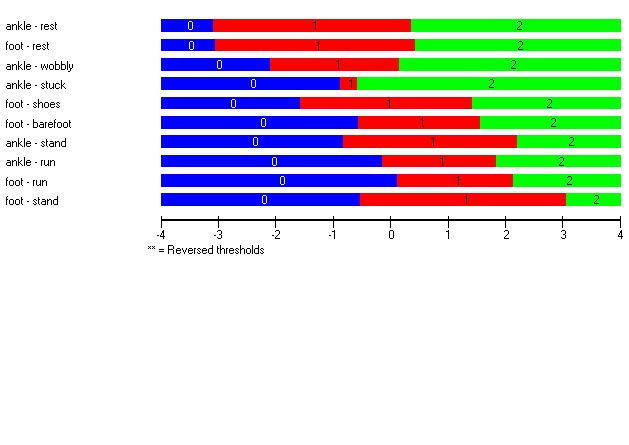 | 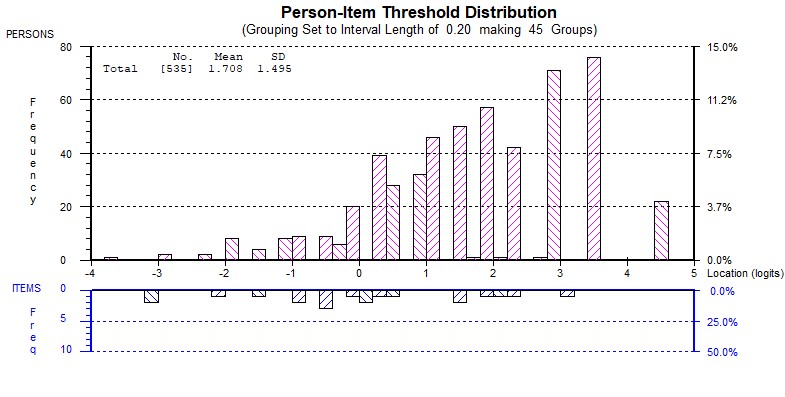 |
| **HIP SYMPTOMS** | |
| 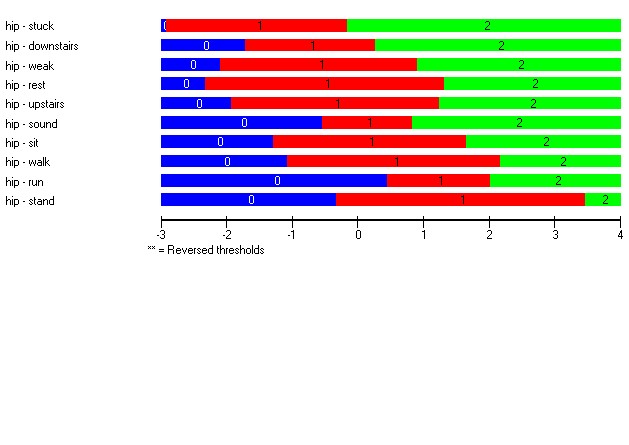 | 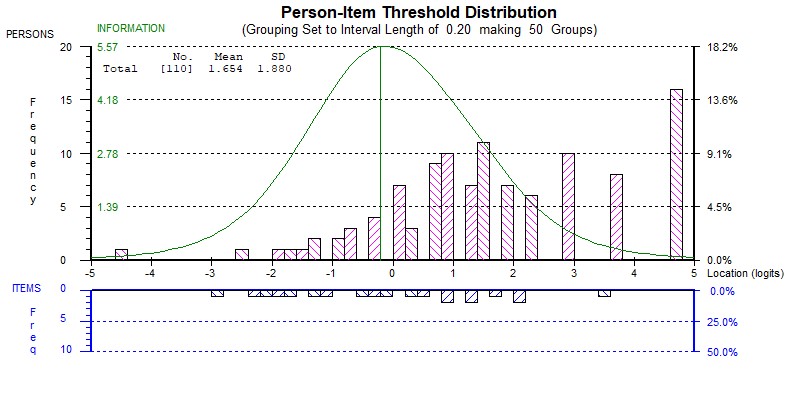 |
| **LEG-RELATED DISTRESS** | |
| 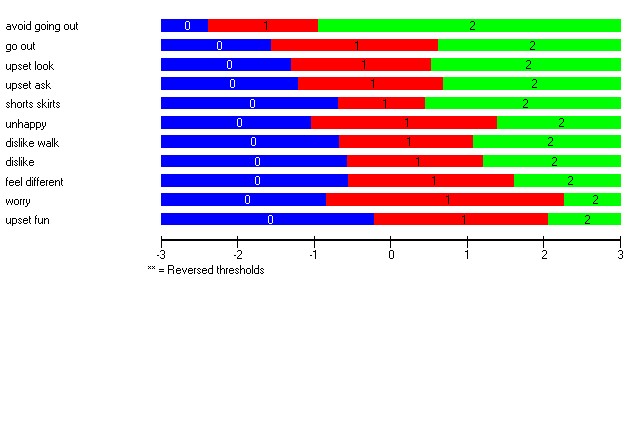 | 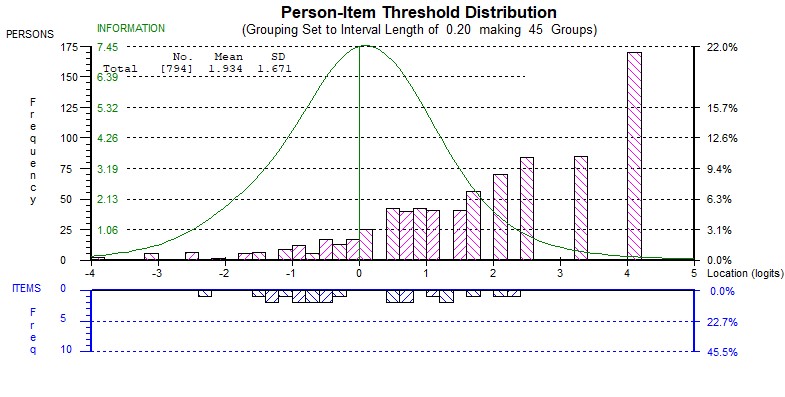 |
| **PSYCHOLOGICALFUNCTION** | |
| 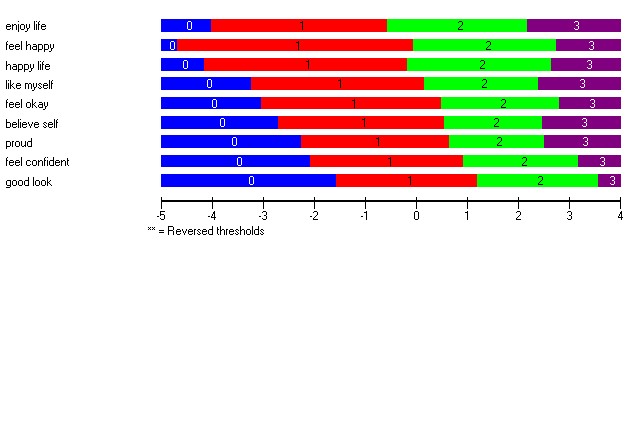 | 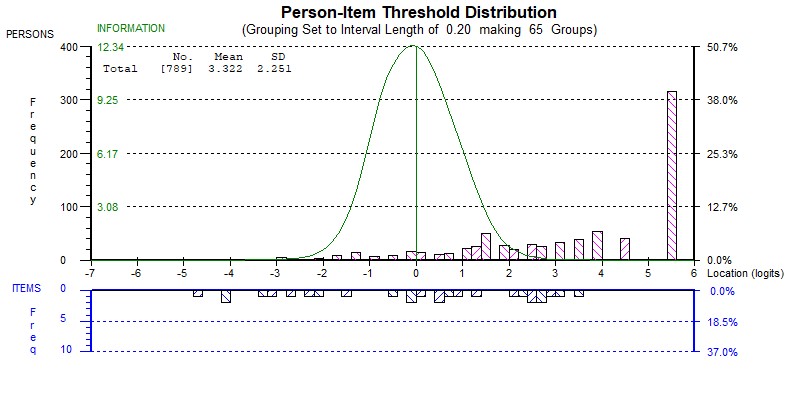 |
| **SOCIAL FUNCTION** | |
| 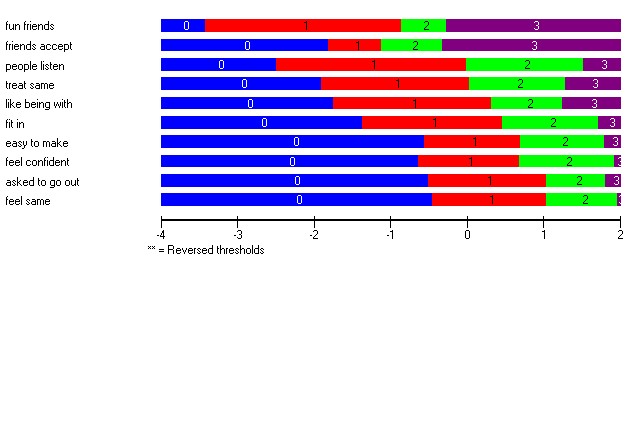 | 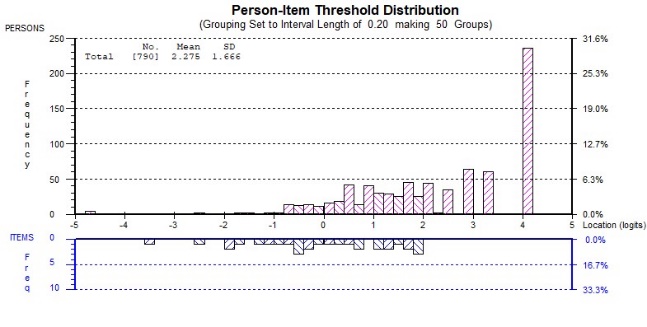 |

**Supplementary Table 4: Construct Validity – Hypothesis 1 & 2**

|  |  | LIMB-Q Kids Physical Function | PedsQL Physical Total | PROMIS Mobility Scale T-score |
| --- | --- | --- | --- | --- |
| PedsQL Physical Total Score | Pearson correlation | 0.71** | -- |  |
|  | Sig. (2-tailed) | <.001 |  |  |
|  | N | 585 | 586 |  |

**Correlation is significant at the 0.01 level (2-tailed)

Hypothesis 1: LIMB-Q Kids Physical Function scale correlates with PEDSQL related >0.5

Hypothesis 2: LIMB-Q Kids Physical Function scale correlates with PROMIS Mobility related >0.5

**Supplementary Table 5: Construct Validity – Hypothesis 3&4**

|  |  | LIMB-Q Kids Social Function | LIMB-Q Kids Psychological Function |
| --- | --- | --- | --- |
| PedsQL Social Total | Pearson correlation | 0.64** | 0.484** |
|  | Sig. (2-tailed) | <.0.001 | <0.001 |
|  | N | 586 | 586 |
| PedsQL Emotion Total | Pearson Correlation | 0.52** | 0.598** |
|  | Sig. (2-tailed) | <.001 | <.0.001 |
|  | N | 586 | 586 |

**Correlation is significant at the 0.01 level (2-tailed)

Hypothesis 3: LIMB-Q Kids Social Function scale correlates with PEDSQL Social Scale related >0.5

Hypothesis 4: LIMB-Q Kids Social Function scale correlates with PEDSQL Emotion Scale related >0.5

**Supplementary Table 6 – Construct Validity – Hypothesis 5**

|  |  | Leg Appearance |
| --- | --- | --- |
| Hip Symptom | Pearson correlation | 0.15** |
|  | Sig. (2-tailed) | <.001 |
|  | N | 784 |
| Leg Symptom | Pearson correlation | 0.27** |
|  | Sig. (2-tailed) | <.001 |
|  | N | 784 |
| Knee Symptom | Pearson Correlation | 0.19** |
|  | Sig. (2-tailed) | <0.001 |
|  | N | 779 |
| Foot and Ankle Symptom | Pearson Correlation | 0.19** |
|  | Sig. (2-tailed) | <0.001 |
|  | N | 755 |

**Correlation is significant at the 0.01 level (2-tailed)

Hypothesis 5: LIMB-Q Kids Leg-Appearance scale should be not related to LIMB-Q Kids symptom scales <0.3

**Supplementary Table 7 – Construct Validity – Hypothesis 6**

|  |  | Leg Appearance |
| --- | --- | --- |
| Leg-related Distress | Pearson correlation | 0.32** |
|  | Sig. (2-tailed) | <.001 |
|  | N | 783 |

** Correlation is significant at the 0.01 level (2-tailed).

Hypothesis 6: LIMB-Q Kids Leg-Appearance and Leg-related Distress scales 0.3-0.5 related but dissimilar

**Supplementary Table 8 – Construct Validity hypothesis 7 & 8**

|  |  | Leg-related Distress |
| --- | --- | --- |
| Social Function | Pearson correlation | 0.48** |
|  | Sig. (2-tailed) | <0.001 |
|  | N | 789 |
| Psychological Function | Pearson Correlation | 0.49** |
|  | Sig. (2-tailed) | <0.001 |
|  | N | 788 |

** Correlation is significant at the 0.01 level (2-tailed).

Hypothesis 7: LIMB-Q Kids Leg-related Distress scales correlate with LIMB-Q Kids Psychological Function scale >0.5

Hypothesis 8: LIMB-Q Kids Leg-related Distress scale correlates with LIMB-Q Kids Social Function scale 0.3-0.5

**Supplementary Table 9- Construct Validity- Hypothesis 9**

|  |  | Leg Appearance |
| --- | --- | --- |
| Social Function | Pearson correlation | 0.29** |
|  | Sig. (2-tailed) | <0.001 |
|  | N | 782 |
| Psychological Function | Pearson Correlation | 0.23** |
|  | Sig. (2-tailed) | <0.001 |
|  | N | 781 |

** Correlation is significant at the 0.01 level (2-tailed).

Hypothesis 9: LIMB-Q Kids Leg-Appearance, Social Function, and Psychological Function scales will correlate 0.3-0.5

**Supplementary Table 10 – Construct Validity – Hypothesis 10**

|  |  | Physical Function |
| --- | --- | --- |
| Leg-related Distress | Pearson correlation | 0.41** |
|  | Sig. (2-tailed) | <0.001 |
|  | N | 794 |

** Correlation is significant at the 0.01 level (2-tailed).

Hypothesis 10: LIMB-Q Kids Physical Function and Leg-related Distress scales are related but dissimilar 0.3-05

**Supplementary Table 11 – Construct Validity, Hypothesis 11**

|  |  | N | Mean | Std. Deviation | Std. Error | 95% Confidence Interval for Mean | | Sig |
| --- | --- | --- | --- | --- | --- | --- | --- | --- |
|  |  |  |  |  |  | Lower Bound | Upper Bound |  |
| Physical Function | Normal | 128 | 76.08 | 23.63 | 2.09 | 71.94 | 80.21 | <0.001 |
|  | Minimal complexity | 527 | 75.45 | 22.45 | 0.98 | 73.53 | 77.37 |  |
|  | Moderate complexity | 115 | 59.86 | 25.08 | 2.34 | 55.23 | 64.49 |  |
|  | High/Substantial complexity | 29 | 52.28 | 19.11 | 3.55 | 45.01 | 59.54 |  |
| Leg Appearance | Normal | 125 | 61.57 | 29.25 | 2.62 | 56.39 | 66.75 | <0.001 |
|  | Minimal complexity | 517 | 53.57 | 27.60 | 1.21 | 51.19 | 55.96 |  |
|  | Moderate complexity | 114 | 47.56 | 24.73 | 2.32 | 42.97 | 52.15 |  |
|  | High/Substantial complexity | 28 | 46.14 | 22.44 | 4.24 | 37.44 | 54.85 |  |
| Hip Symptom | Normal | 127 | 85.81 | 16.62 | 1.48 | 82.89 | 88.73 | 0.005 |
|  | Minimal complexity | 526 | 82.86 | 19.92 | 0.87 | 81.15 | 84.56 |  |
|  | Moderate complexity | 115 | 79.17 | 18.95 | 1.77 | 75.67 | 82.67 |  |
|  | High/Substantial complexity | 29 | 73.93 | 20.08 | 3.73 | 66.29 | 81.57 |  |
| Leg Symptoms | Normal | 128 | 72.58 | 20.15 | 1.78 | 69.05 | 76.10 | <0.001 |
|  | Minimal complexity | 527 | 67.51 | 19.75 | 0.86 | 65.82 | 69.20 |  |
|  | Moderate complexity | 115 | 60.47 | 17.57 | 1.64 | 57.22 | 63.72 |  |
|  | High/Substantial complexity | 29 | 57.45 | 16.64 | 3.09 | 51.12 | 63.78 |  |
| Knee Symptoms | Normal | 127 | 83.24 | 18.41 | 1.63 | 80.00 | 86.47 | <0.001 |
|  | Minimal complexity | 524 | 79.04 | 19.43 | 0.85 | 77.37 | 80.70 |  |
|  | Moderate complexity | 112 | 73.36 | 19.14 | 1.81 | 69.77 | 76.94 |  |
|  | High/Substantial complexity | 29 | 69.72 | 19.32 | 3.59 | 62.38 | 77.07 |  |
| Foot &Ankle Symptoms | Normal | 119 | 83.19 | 16.44 | 1.51 | 80.21 | 86.18 | 0.003 |
|  | Minimal complexity | 513 | 78.30 | 19.90 | 0.88 | 76.57 | 80.02 |  |
|  | Moderate complexity | 109 | 74.26 | 21.51 | 2.06 | 70.17 | 78.34 |  |
|  | High/Substantial complexity | 26 | 72.04 | 23.86 | 4.68 | 62.40 | 81.67 |  |
| Leg-related Distress | Normal | 127 | 81.94 | 17.36 | 1.54 | 78.90 | 84.99 | <0.001 |
|  | Minimal complexity | 523 | 73.97 | 19.86 | 0.87 | 72.26 | 75.68 |  |
|  | Moderate complexity | 115 | 65.54 | 21.48 | 2.00 | 61.57 | 69.51 |  |
|  | High/Substantial complexity | 29 | 62.86 | 21.38 | 3.97 | 54.73 | 71.00 |  |
| Social Function | Normal | 127 | 81.51 | 18.22 | 1.62 | 78.31 | 84.71 | 0.001 |
|  | Minimal complexity | 520 | 77.47 | 19.98 | 0.88 | 75.74 | 79.19 |  |
|  | Moderate complexity | 114 | 74.00 | 19.36 | 1.81 | 70.41 | 77.59 |  |
|  | High/Substantial complexity | 29 | 67.93 | 20.46 | 3.80 | 60.15 | 75.71 |  |
| Psychological Function | Normal | 127 | 82.13 | 18.75 | 1.66 | 78.84 | 85.43 | 0.046 |
|  | Minimal complexity | 520 | 81.59 | 20.48 | 0.90 | 79.82 | 83.35 |  |
|  | Moderate complexity | 113 | 76.87 | 21.65 | 2.04 | 72.83 | 80.90 |  |
|  | High/Substantial complexity | 29 | 74.48 | 27.76 | 5.15 | 63.92 | 85.04 |  |

Hypothesis 11: With increased severity based on the LLRS-AIM index, LIMB-Q Kids scale scores will decrease

**Supplementary Table 12 – Construct Validity, Hypothesis 12**

|  | Does this patient have physical symptoms related to Knee | N | Mean | Std. Deviation | Std. Error Mean | Sig |
| --- | --- | --- | --- | --- | --- | --- |
| Hip Symptoms | No Symptoms | 687 | 84.84 | 18.20 | 0.69 | <0.001 |
|  | Symptoms | 110 | 67.67 | 20.37 | 1.94 |  |

|  | Does this patient have physical symptoms related to the Leg overall | N | Mean | Std. Deviation | Std. Error Mean | Sig |
| --- | --- | --- | --- | --- | --- | --- |
| Leg Symptoms | No Symptoms | 506 | 70.11 | 20.32 | 0.90 | <0.001 |
|  | Symptoms | 293 | 61.47 | 17.46 | 1.02 |  |

|  | Does this patient have physical symptoms related to Knee | N | Mean | Std. Deviation | Std. Error Mean | Sig |
| --- | --- | --- | --- | --- | --- | --- |
| Knee Symptoms | No Symptoms | 574 | 82.23 | 18.02 | 0.75 | <0.001 |
|  | Symptoms | 218 | 68.91 | 19.82 | 1.34 |  |

|  | Does this patient have physical symptoms related to Foot and ankle | N | Mean | Std. Deviation | Std. Error Mean | Sig |
| --- | --- | --- | --- | --- | --- | --- |
| Foot and Ankle Symptoms | No Symptoms | 644 | 80.68 | 19.02 | 0.75 | <0.001 |
|  | Symptoms | 123 | 65.67 | 19.96 | 1.80 |  |

Hypothesis 12: If the clinical team reported symptoms for a body part score on the corresponding symptom scale will be lower

**Supplementary Table 13 – Construct Validity Hypothesis 13**

|  |  | N | Mean | Std. Deviation | Std. Error | 95% Confidence Interval for Mean | | Sig |
| --- | --- | --- | --- | --- | --- | --- | --- | --- |
|  |  |  |  |  |  | **Lower Bound** | **Upper Bound** |  |
| Physical Function | One deformity | 304 | 76.29 | 21.82 | 1.25 | 73.83 | 78.75 | <0.001 |
|  | Two deformities | 125 | 67.24 | 26.33 | 2.36 | 62.58 | 71.90 |  |
|  | Three deformities | 31 | 60.26 | 24.40 | 4.38 | 51.31 | 69.21 |  |
|  | More than three deformities | 24 | 54.46 | 22.66 | 4.63 | 44.89 | 64.03 |  |
| Leg Appearance | One deformity | 297 | 54.19 | 28.76 | 1.67 | 50.91 | 57.48 | 0.021 |
|  | Two deformities | 124 | 50.44 | 22.78 | 2.05 | 46.39 | 54.49 |  |
|  | Three deformities | 30 | 38.53 | 26.30 | 4.80 | 28.71 | 48.35 |  |
|  | More than three deformities | 24 | 52.50 | 26.19 | 5.35 | 41.44 | 63.56 |  |
| Hip Symptoms | One deformity | 304 | 84.21 | 19.07 | 1.09 | 82.06 | 86.36 | 0.027 |
|  | Two deformities | 125 | 79.65 | 20.91 | 1.87 | 75.95 | 83.35 |  |
|  | Three deformities | 31 | 76.16 | 22.75 | 4.09 | 67.82 | 84.51 |  |
|  | More than three deformities | 24 | 77.92 | 17.10 | 3.49 | 70.70 | 85.14 |  |
| Leg Symptoms | One deformity | 304 | 68.32 | 19.48 | 1.12 | 66.12 | 70.52 | 0.007 |
|  | Two deformities | 125 | 63.78 | 20.22 | 1.81 | 60.20 | 67.36 |  |
|  | Three deformities | 31 | 59.94 | 19.31 | 3.47 | 52.85 | 67.02 |  |
|  | More than three deformities | 24 | 58.88 | 14.72 | 3.01 | 52.66 | 65.09 |  |
| Knee Symptoms | One deformity | 303 | 79.70 | 19.31 | 1.11 | 77.51 | 81.88 | 0.009 |
|  | Two deformities | 124 | 73.40 | 19.54 | 1.75 | 69.92 | 76.87 |  |
|  | Three deformities | 31 | 72.48 | 21.64 | 3.89 | 64.55 | 80.42 |  |
|  | More than three deformities | 24 | 75.54 | 16.31 | 3.33 | 68.65 | 82.43 |  |
| Foot and Ankle Symptoms | One deformity | 294 | 78.28 | 19.49 | 1.14 | 76.04 | 80.52 | 0.054 |
|  | Two deformities | 124 | 73.35 | 22.19 | 1.99 | 69.41 | 77.30 |  |
|  | Three deformities | 30 | 71.83 | 25.97 | 4.74 | 62.14 | 81.53 |  |
|  | More than three deformities | 23 | 71.70 | 20.33 | 4.24 | 62.91 | 80.49 |  |
| Leg-related Distress | One deformity | 301 | 75.55 | 20.23 | 1.17 | 73.25 | 77.84 | <0.001 |
|  | Two deformities | 125 | 66.72 | 21.79 | 1.95 | 62.86 | 70.58 |  |
|  | Three deformities | 31 | 65.90 | 20.80 | 3.74 | 58.27 | 73.53 |  |
|  | More than three deformities | 24 | 65.42 | 18.54 | 3.79 | 57.59 | 73.25 |  |

Hypothesis 13: With the increase in the number of deformities on the LLRS AIM index, LIMB-Q Kids scale scores decrease

**Supplementary Table 14- Construct Validity – Hypothesis 14**

|  | Frame status | N | Mean | Std. Deviation | Std. Error Mean | Sig |
| --- | --- | --- | --- | --- | --- | --- |
| Physical Function | Currently have frame | 58 | 51.40 | 21.04 | 2.76 | <0.001 |
|  | Frame removed >6m | 91 | 71.81 | 21.56 | 2.26 |  |
| Leg Appearance | Currently have frame | 56 | 41.61 | 25.61 | 3.42 | 0.013 |
|  | Frame removed >6m | 89 | 52.30 | 24.59 | 2.61 |  |

Hypothesis 14: Patients in frame report lower scores on LIMB-Q Kids scales as compared to patients who are > 6 months post-frame removal

**Supplementary Table 15 – Construct Validity – Hypothesis 15**

|  |  | N | Mean | Std. Deviation | Std. Error | 95% Confidence Interval for Mean | |  |
| --- | --- | --- | --- | --- | --- | --- | --- | --- |
|  |  |  |  |  |  | **Lower Bound** | **Upper Bound** | **Sig** |
| Leg Appearance | Not at all | 139 | 41.36 | 25.63 | 2.17 | 37.06 | 45.66 | <0.001 |
|  | A little bit | 168 | 51.08 | 24.34 | 1.88 | 47.37 | 54.78 |  |
|  | Quite a bit | 140 | 55.89 | 23.59 | 1.99 | 51.94 | 59.83 |  |
|  | Very much | 133 | 71.00 | 26.73 | 2.32 | 66.42 | 75.58 |  |
| Leg-related Distress | Not at all | 140 | 64.20 | 21.68 | 1.83 | 60.58 | 67.82 | <0.001 |
|  | A little bit | 168 | 69.25 | 19.35 | 1.49 | 66.30 | 72.20 |  |
|  | Quite a bit | 140 | 75.53 | 17.42 | 1.47 | 72.62 | 78.44 |  |
|  | Very much | 133 | 82.80 | 18.86 | 1.64 | 79.57 | 86.04 |  |
| Social Function | Not at all | 140 | 73.24 | 18.98 | 1.60 | 70.06 | 76.41 | <0.001 |
|  | A little bit | 169 | 75.28 | 19.95 | 1.53 | 72.26 | 78.31 |  |
|  | Quite a bit | 139 | 77.86 | 17.94 | 1.52 | 74.85 | 80.87 |  |
|  | Very much | 133 | 83.64 | 19.49 | 1.69 | 80.30 | 86.98 |  |
| Psychological Function | Not at all | 139 | 77.69 | 22.72 | 1.93 | 73.88 | 81.50 | <0.001 |
|  | A little bit | 169 | 77.77 | 21.18 | 1.63 | 74.55 | 80.99 |  |
|  | Quite a bit | 139 | 81.92 | 19.08 | 1.62 | 78.72 | 85.12 |  |
|  | Very much | 133 | 87.22 | 18.28 | 1.58 | 84.08 | 90.35 |  |

*For participants reporting liking their scars, LIMB-Q Kids scale scores increase with the increase in the likeness.*

*Item: How much do you like how the scars on your leg looks?*

Hypothesis 15: For participants reporting liking their scars, LIMB-Q Kids scale scores increase with increase in the likeness

**Supplementary Table 16– Construct Validity – Hypothesis** 16

|  |  | N | Mean | Std. Deviation | Std. Error | 95% Confidence Interval for Mean | |  |
| --- | --- | --- | --- | --- | --- | --- | --- | --- |
|  |  |  |  |  |  | **Lower Bound** | **Upper Bound** | **Sig** |
| Leg Appearance | Not at all | 155 | 17.22 | 15.78 | 1.27 | 14.71 | 19.72 | <0.001 |
|  | A little bit | 182 | 42.60 | 11.01 | 0.82 | 40.99 | 44.22 |  |
|  | Quite a bit | 189 | 56.85 | 9.53 | 0.69 | 55.48 | 58.21 |  |
|  | Very much | 257 | 81.48 | 18.36 | 1.15 | 79.23 | 83.74 |  |
| Leg-related Distress | Not at all | 155 | 68.90 | 25.31 | 2.03 | 64.89 | 72.92 | <0.001 |
|  | A little bit | 181 | 62.52 | 15.41 | 1.15 | 60.26 | 64.78 |  |
|  | Quite a bit | 189 | 74.56 | 17.90 | 1.30 | 71.99 | 77.12 |  |
|  | Very much | 257 | 83.77 | 16.72 | 1.04 | 81.72 | 85.82 |  |

*For participants reporting liking the appearance of their leg, LIMB-Q Kids scale scores increase with the increase in the likeness.*

*Item: How much do you like how your leg looks overall?*

Hypothesis 16: For participants reporting liking the appearance of their leg, LIMB-Q Kids scale scores increase with the increase in the likeness
